# Seismic vulnerability assessment to earthquake at urban scale: A case of Mostaganem city in Algeria

**DOI:** 10.4102/jamba.v10i1.473

**Published:** 2018-03-27

**Authors:** Belkacem Chaibedra, Abdelkader Benanane, Zohra Boutaraa

**Affiliations:** 1 Laboratory of Materials and Constructions Processes, Abdelhamid Ibn Badis University, Algeria; 2 Materials Sciences and Environment Laboratory, Hassiba Ben Bouali University, Algeria

## Abstract

The focus of this study was the seismic vulnerability assessment of buildings constituting Mostaganem city in Algeria. Situated 320 km to the west of Algiers, Mostaganem city encompasses a valuable cultural and architectural built heritage. The city has suffered several moderate earthquakes in recent years; this has led to extensive structural damage to old structures, especially unreinforced historical buildings. This study was divided into two essential steps, the first step being to establish fragility curves based on a non-linear static pushover analysis for each typology and height of buildings. Twenty-seven pushover analyses were performed by means of SAP2000 software (three analyses for each type of building). The second step was to adopt the US HAZUS software and to modify it to suit the typical setting and parameters of the city of Mostaganem. A seismic vulnerability analysis of Mostaganem city was conducted using HAZUS software after inputting the new parameters of the fragility curves established within the first step. The results indicated that the number of poor-quality buildings expected to be totally destroyed under a 5.5 Mw earthquake scenario could reach more than 28 buildings. Three percent of unreinforced masonry (URM) buildings were completely damaged and 10% were extensively damaged. Of the concrete frame buildings, 6% were extensively damaged and 19% were moderately damaged. According to the built year, 6% of both concrete frame and URM buildings built before 1980 are estimated to be collapsing. Buildings constructed between 1980 and 1999 are more resistant; 8% of those structures were extensively damaged and 18% were moderately damaged. Only 10% of buildings constructed after 1999 were moderately damaged. The results also show that the main hospital of the city, built before 1960, will be extensively damaged during an earthquake of 5.5 Mw. The number of human casualties could reach several hundreds – 10.5% of residents of URM buildings are injured or dead. Compared with the URM buildings, concrete frame buildings have lower casualty rates of 1.5% and 0.5% for those built before and after 1980, respectively. It was concluded that Mostaganem city belongs to seismic vulnerable zones in Algeria; in this regard, an action plan is needed for the rehabilitation of old constructions. In addition, the effectiveness of establishing and introducing new and appropriate fragility curves was demonstrated.

## Introduction

The recent tragic earthquake events which hit some regions of Algerian territory such as Chlef, Boumerdes and Algiers (Ayadi & Bezzeghoud 2014) are a living testimony of the state of non-seismic protection for many municipalities in Algeria. This is owing to a series of hazardous factors such as the age of the buildings, the poor quality of the structural systems and the insufficient maintenance of buildings. The seismic vulnerability assessment of an urban environment characterises the ability of buildings and structures to support horizontal forces when earthquake events occur. Reducing the seismic risk requires an assessment of the physical seismic vulnerability of buildings to earthquake hazards. This axis of earthquake engineering experienced a development of major projects in some countries and assessment tools based on the Geographic Information System (GIS). This was developed in the framework of major international programmes, principally in seismic-prone countries such Greece, Romania, Italy and the United States. For instance, the Risk Assessment Tools for Diagnosis of Urban Areas against Seismic Disasters (RADIUS) was launched by the International Decade for Natural Disaster Reduction (IDNDR) in 1996 and implemented in nine cities on different continents (Okazaki et al. 2000). The RISK-UE project was another advanced approach to evaluating seismic risk, with application in seven European cities. The main objectives were the development of a general methodology for seismic risk assessment of European cities (Milutinovic & Trendafiloski 2003). In the United States, HAZUS is a software package developed by the Federal Emergency Management Agency (HAZUS99 & FEMA 1999), used mainly in instances of natural disasters like earthquakes, floods and hurricanes. In recent years, a significant number of researchers have focused on loss assessment for earthquake-prone regions using the HAZUS framework. For instance, in 2014, Wei et al. (2014) investigated the economic feasibility of the building retrofit by means of a cost–benefit analysis applied in Tiberias in Israel with HAZUS Tools. In 2015, Yu et al. (2016) investigated the sensitivity of damage estimation results to epistemic uncertainty related to three different attenuation functions.

HAZUS contains a variety of default parameters and databases. We can run a loss estimation analysis using only default data, but the results would be subject to a great deal of uncertainty. Within the main important default parameters, data that should be modified are those with the capacity and fragility curves that can be obtained through non-linear static analysis (pushover), used with capacity spectrum method (CSM) techniques (Barbat et al. 2012). A building-capacity curve is a plot of a building’s lateral load resistance as a function of a characteristic lateral displacement. It is derived from a plot of static-equivalent base shear versus building displacement. In order to facilitate a direct comparison with earthquake demand that is overlaying the capacity curve with a response spectrum, the force (base shear) axis is converted to spectral acceleration and the displacement axis is converted to spectral displacement. Such a plot would provide an estimate of the building’s ‘true’ deflection (displacement response) for any given earthquake response spectrum. From the results of a pushover analysis, fragility curves can be plotted by calculating the median and the standard deviation of each specific curve (Vargas et al. 2013).

A complete seismic assessment requires the analysis of all components implicated in a seismic event; that is, building stock, transportation system, infrastructure system and critical facilities (HAZUS99 & FEMA 1999). But the compilation of such data is often costly and time-consuming. Therefore, this work focuses on the physical damage of building stock in Mostaganem city and its corresponding casualties.

## The study region

**FIGURE 1:**
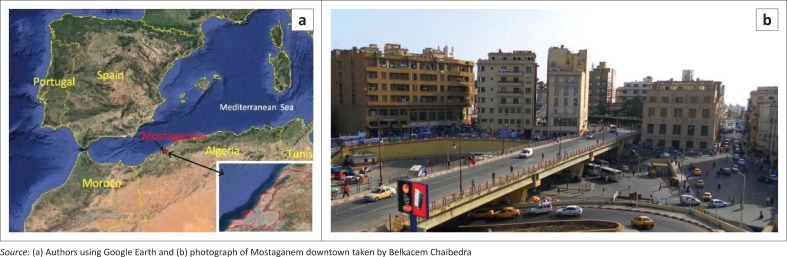
Location of Mostaganem city and a photograph of Mostaganem downtown. (a) Geographical location of Mostaganem city and (b) photograph of Mostaganem downtown.

The city of Mostaganem is located on the Mediterranean Sea, in the northwestern region of Algeria. It was founded in the 11th century, under the name Murustage, but was overrun in 1516 by Barbarossa. Even though Barbarossa was a pirate, he managed to transform Mostaganem into a lucrative pirate port (De la Primaudaie 1861), and a port for commercial traffic. In the 1700s, the Ottoman Empire took over the seaport, and soon the city fell into decline. The French were the last foreign rulers of Mostaganem; they took control of the city in 1833 until Algeria declared its independence in 1962. However, the city of Mostaganem has become a popular tourist attraction. It has remained a fishing port, with an estimated population of approximately 145 696 residents (ONS 2011). Figure 1 shows the location of Mostaganem city in the Mediterranean Sea and a photograph of Mostaganem downtown.

### Building inventory

**FIGURE 2:**
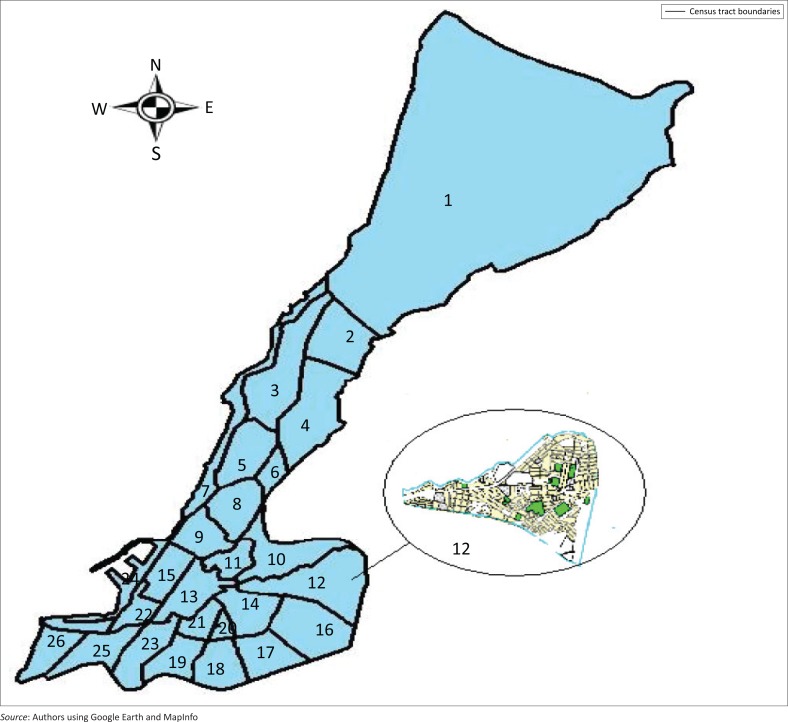
The 21 census tracts of Mostaganem city.

In developing a regional inventory, it is roughly impractical from a cost point of view to identify and inventory each man-made construction separately. Some essential structures such as hospitals, schools, emergency operation centres and fire stations may be identified individually, but the majority of buildings in a region are grouped collectively and identified by their total value or square footage. To allow the modelling of spatial distinction in types and occupancies of buildings, a region is built up from subregions, and the inventory is collected for each subregion. Mostaganem city is divided into 26 census tracts (Figure 2) that are used as the basic subregion unit, and all the regions are built up by aggregating census tracts. Thus, for each census tract, the inventory of buildings in the study area was carried out, and there were approximately 73 250 dwellings.

For each building, the following characteristics are considered to be introduced in HAZUS:

- building type (house, building, precarious)
- building use (dwelling, educational …)
- age of building – code era
- number of floors and square footage
- structural system
- state of preservation.

**FIGURE 3:**
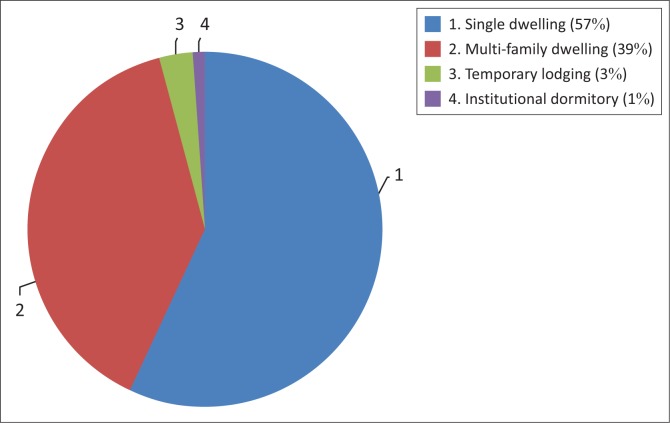
Distribution of residential buildings.

The inventory data regarding the distribution of residential buildings in terms of types of habitations are shown in Figure 3.

After the laboratory team performed a detailed building inventory of the city, using statistical processing of data, buildings within each census tract were aggregated and categorised as the following: (1) According to the occupancy classes and structural types, knowing that HAZUS methodology regroups 36 model building types and 33 occupancy classes and (2) according to the time of construction as (a) before 1980, (b) between 1980 and 1999 and (c) after 1999. The classification according to building type gives the following percentages: 15% are residential buildings and 65% are single–family dwellings. Data show that 70% of the buildings in the study area have 1–3 floors. Buildings constructed before 1980 represent 60% of the total, which are characterised by a significant number of masonry buildings and a low percentage of reinforced concrete buildings constructed without a seismic code design. Twenty-five percent of the buildings were built between 1980 and 1999, and the majority of these buildings are reinforced concrete buildings that have been designed according to the first Algerian seismic code, established in 1981. In 1999, the Algerian seismic code was improved; therefore, buildings constructed in this period are characterised by a good seismic performance which represents 15% of the total buildings.

### Demography

The local demographic data were collected in order to assemble a detailed distribution map of the population for different occupancy types in various time frames. According to the general census of the population in 2008, the population of the town of Mostaganem is estimated at 145 696 inhabitants. Like the Algerian population, the population of the city is young; nearly 36% are aged less than 20 years. The age range between 20 and 59 years represents more than half of the population of the city. Consequently, the population aged 60 and over is very low, only 8.13% of the total population of the city (ONS 2011).

### Seismic activity in the study region

Northern Algeria has been the site of various historical earthquakes and has been subjected to extensive damage, which has accounted for several thousands of victims in the past. Earthquakes up to a magnitude of MS = 7.3 have been recorded in the Ibero–Maghrebian region during the last century, such as the ‘El Asnam’ earthquake of 10 October 1980. Other damaging earthquakes that have affected northern Algeria are the ‘Ain Temouchent’ earthquake on 22 December 1999 (m_bLg_ = 5.7) (Yelles-Chaouche et al. 2004) and the earthquake in ‘Beni Ourtilane’ on 10 November 2000 (m_bLg_ = 5.6) (Montilla, Hamdache & Casado 2003). The study area belongs to the westernmost part of the Chelif Neogene basin, a part of the Tell Atlas chain of Algeria. This area comes under the compressional tectonics range, resulting from 3 mm/yr to 6 mm/yr convergence between the African and Eurasian plates (Mortgat & Shah 1978). Consequently, numerous active reverse faults blind or not have been identified in the region (Benouar 1993; Meghraoui 1988). Mostaganem has not been recognised as a high seismic zone and has not been fully recognised as being exposed to a high risk of significant losses in spite of its vulnerable built environment and high exposure of the population to seismic hazards. Therefore, compared with other regions with elevated seismic activity but with a well-organised risk management strategy, a possible major tremor at Mostaganem could result in extensive damage owing to its relative lack of preparation for seismic events; this was clearly demonstrated on 22 May 2014 at Bouguirat (20 km from Mostaganem), which was subjected to an earthquake of a magnitude of 5.2 Mw. This moderate seismic event was located in a region characterised by low seismic activity where few historical events have been observed. This earthquake showed a maximum intensity of VIII on the Medvedev–Sponheuer–Karnik scale (MSK) and caused extensive damage. In the epicentral region, 1987 houses and eight public buildings were damaged in the urban area as well as 2300 houses in rural areas (Farrugia, Agius & D’Amico 2015). Therefore, a standardised and straightforward risk assessment methodology is needed for public authorities in Mostaganem city to assess the potential seismic risks and to chart necessary risk improvement plans for protecting people’s lives and property. In Table 1, we report the last seven earthquakes that affected Mostaganem.

**TABLE 1:** The last seven earthquakes that occurred at Mostaganem.

Date	Latitude	Longitude	Z (km)	Magnitude
2015	36.043 °N	0.424 °E	13.00	MW 4.2
2014	35.720 °N	0.152 °E	15.00	MW 4.1
2014	35.785 °N	0.241 °E	10.00	MW 4.8
2008	36.514 °N	0.083 °E	0.00	MW 3.4
2008	35.704 °N	0.207 °E	0.00	MW 3.8
2007	35.922 °N	0.063 °E	10.00	MW 4.2
2005	36.251 °N	0.398 °E	0.00	MW 3.3

## Methodology and application

**FIGURE 4:**
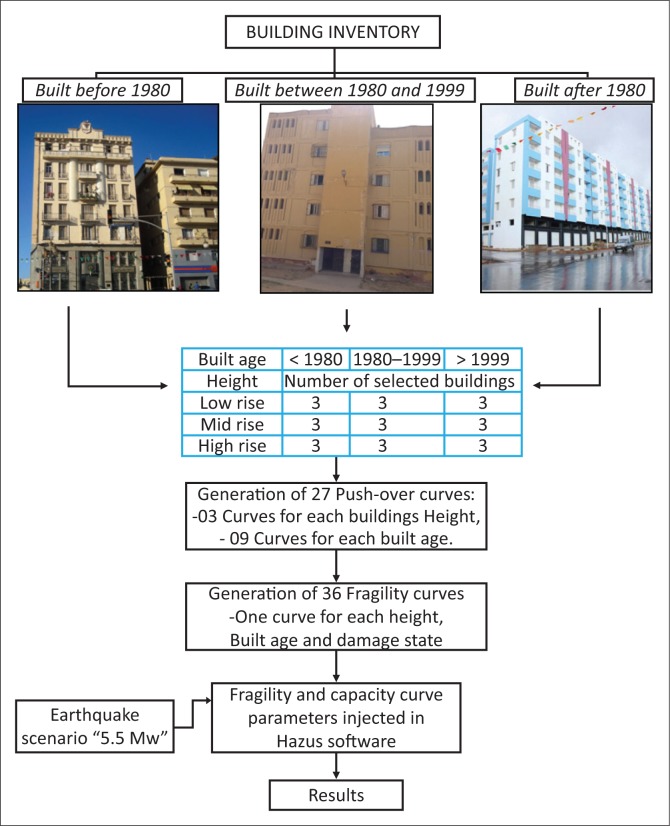
Flowchart of procedure adopted in this study.

The proposed methodology is presented step by step in the flowchart (Figure 4).

### Establishment of fragility curves

#### Bilinear representation of a capacity curve

The pushover analysis technique (APA) has been used in this study to calculate the pushover curve of the structure. It is then transformed in the capacity spectrum by means of the procedure proposed in the ATC-40 (1996). The capacity spectrum is represented in spectral acceleration and spectral displacement coordinates (*sa-sd*) and is used in its simplified bilinear form, defined by the yielding point (*Dy, Ay*) and the ultimate capacity point (*Du, Au*).

#### Damage states

To obtain the damage state thresholds and the corresponding fragility curves, we use simplified methods in order to analyse the expected damage; four damage states have been considered, namely (1) *slight*, (2) *moderate*, (3) *severe* and (4) *extensive-to-collapse*. Table 2 shows the formulae adopted for the calculation of damage state thresholds.

**TABLE 2:** Damage state thresholds.

Damage state	Damage state thresholds
Slight	*S*_*d1*_ = *0.7* * *Dy*
Moderate	*S*_*d2*_ = *Dy*
Severe	*S*_*d3*_ = *Dy+0.25(Du – Dy)*
Complete	*S*_*d4*_ = *Du*

**FIGURE 5:**
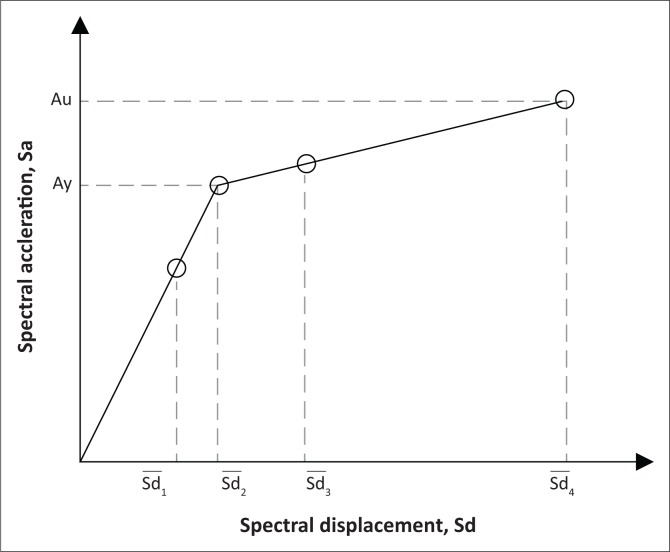
Bilinear representation of capacity curve and the corresponding damage state.

For a given damage state, according to the suggestion considered in the RISK-UE project (Milutinovic & Trendafiloski 2003), the damage state threshold is defined by the 50% probability of incidence. This damage state threshold can be defined in the next basic approach from the bilinear capacity spectrum. Figure 5 represents the position of each damage state threshold on the capacity curve.

#### Fragility curves

**FIGURE 6:**
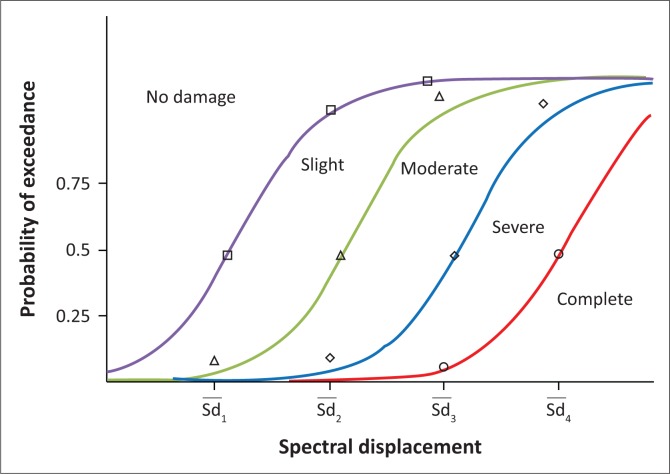
Construction of fragility curves.

For a specified damage state, *Sd*_*i*_, the fragility curve defines the probability that *Sd*_*i*_ be equalled or exceeded as a function of a parameter defining the intensity of the seismic action (HAZUS99 & FEMA 1999). The following equation defines the fragility curve as a function of *Sd*:

$$\text{P}[\text{ds}/\text{Sd}]=\phi \left[\frac{1}{\beta \text{dsi}}\text{ln}\left(\frac{Sdi}{Sd,dsi}\right)\right]$$ [Eqn 1]

where *ϕ* stands for the cumulative lognormal distribution, *ds* is the expected damage, *Sd* is the spectral displacement and *S_d,dsi_* and *β*_dsi_ are the median values and standard deviations of the corresponding normal distributions. For each capacity curve, we calculate the four average displacement values in which the building reaches the limit of damage state (S_d1_, S_d2_, S_d3_, S_d4_). The values of parameters βdsi were determined directly from the HAZUS software. Figure 6 shows the representation of fragility curves that correspond to each damage state.

### HAZUS methodology

The HAZUS earthquake loss estimation method consists of numerous components with various acting as inputs of others. The fundamental inputs of these components are Potential Earth Science Hazards (PESH) and building inventories, from which a full analysis can be run to offer an evaluation in the subsequent fields: direct and induced physical damage; and direct and indirect economic and/or social damage. The HAZUS software package, developed by US FEMA, is a free standardised GIS-based risk assessment tool for hazard analysis and has been widely validated for its applicability in the United States (Montilla et al. 2003). Owing to limited accessibility to some of the data, the Mostaganem study only used part of the analysis models in HAZUS, including ground motion and ground failure predictions, direct physical damage of general building stock and essential facility, and casualty estimation. Despite the fact that HAZUS was originally designed for use in the United States, this standardised seismic risk estimation software has been adopted and validated worldwide because of its merit of being suitable for modification in international settings (Wei et al. 2014; Yu, Chouinard & Rosset 2016). The possibility of supplanting the databases and modifying the default functions with local parameters forms the basis for the application of HAZUS to an international setting. Therefore, adopting HAZUS for an international, local-scale setting requires the careful performance of a series of operations of each module. The parameters of capacity and fragility curves calculated before were inputted in the software.

### The earthquake scenario

The earthquake scenario simulated in this study is characterised by a magnitude of 5.5 Mw and the location of the epicentre is the same as the earthquake that took place in Mostaganem city on 08 August 2007, with a magnitude of 4.2 Mw. Being the only strong earthquake that affected several structures and the location of its epicentre, which was located inside the city, this event seems to be the best choice for loss estimations calculated by HAZUS for Mostaganem city. As Mostaganem is not recognised as a high seismic zone, the magnitude of the earthquake scenario was amplified and limited at the value of 5.5 Mw. We did not take a higher value of magnitude for two reasons: first, the city is not known as a high seismic zone; and second, because the city has a significant number of older buildings which are highly vulnerable to even medium-magnitude earthquakes.

## Results and discussion

### Building damage

**FIGURE 7:**
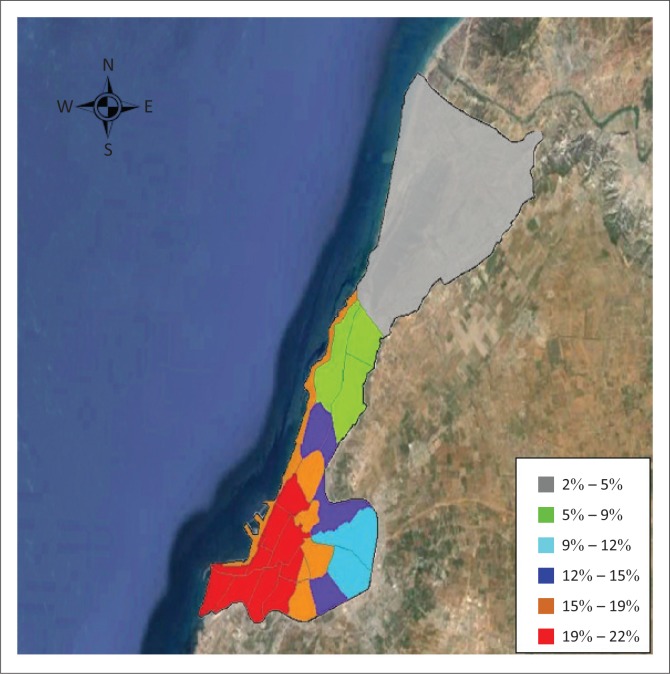
Extensively damaged buildings.

Five categories are defined in HAZUS for building damage: no damage, slight damage, moderate damage, extensive damage and complete damage. The number of damaged buildings is converted from the probability of damage to the buildings for each building type. The results show that for an earthquake of a magnitude of 5.5 Mw, with its epicentre located inside the city, 123 buildings were moderately damaged and 64 were extensively damaged. The number of buildings that are expected to be completely damaged is around 28. The excellent ability of HAZUS to resolve the expected damage by specific census tracts and different damage categories seems to be very promising. A closer look at the epicentral area shows that 24 buildings are expected to be completely destroyed, whereas farther away, only four buildings are expected to collapse. An analysis of the census tract number 22, corresponding to the old Kasbah of ‘Tijdit’ zone, which is 3 km away from the epicentre, shows that the number of damaged buildings is higher than the other places, and this may be because of the age and quality of constructions in this area. Figure 7 shows the distribution of extensively damaged buildings obtained by the seismic simulation on HAZUS software.

#### Building damage by structural type

**FIGURE 8:**
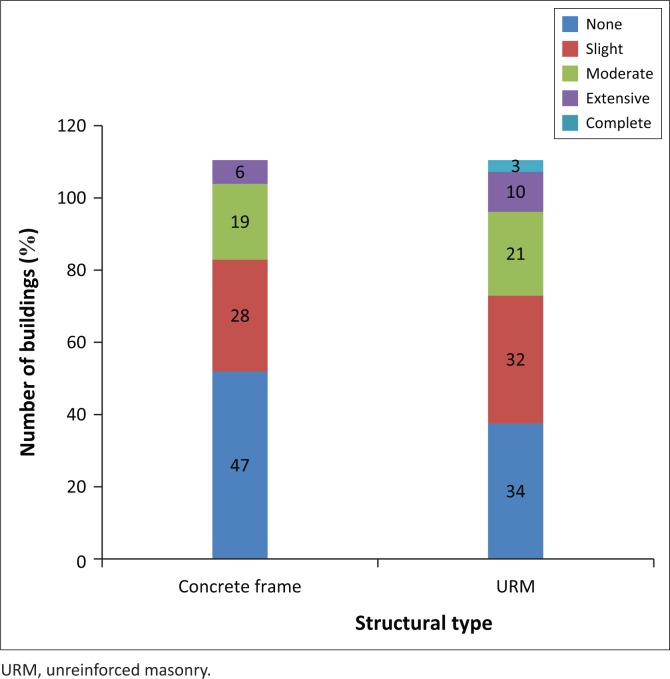
Building damage for unreinforced masonry and concrete frame building types.

Analysing the number of damaged buildings of unreinforced masonry (as depicted in Figure 7), 3% of URM buildings were completely damaged, 10% were extensively damaged and 21% were moderately damaged. For concrete frames, 6% were extensively damaged and 19% were moderately damaged. As expected, the URM is recognised as one of the building types which is most seismically vulnerable. Here, we observe that URM buildings are the most damaged compared with concrete frame buildings; this can be explained by the considerable number of old URM buildings built before 1980 which are vulnerable even to a medium earthquake (5.5 Mw). Figure 8 summarises the percentage of building damage for unreinforced masonry and concrete frame buildings.

#### Building damage by occupancy

In terms of occupancy class, residential buildings account for more than 95% of the total damaged buildings. But that does not mean that the other buildings belonging to other occupancy classes are not affected. The results show that two government buildings were completely damaged, while eight buildings were extensively damaged because there are many old buildings that were built before 1960 and are still used by the authorities. Concerning religious buildings, three mosques were completely damaged and four were extensively damaged; this can also be explained by the built year and structure type of those structures, knowing that 70% of mosques were built with masonry and without rigorous supervision during construction. Table 3 summarises the number of damaged buildings in terms of general occupancy.

**TABLE 3:** Building damage by general occupancy.

Building class	Slight	Moderate	Extensive	Complete
Residential	312	90	44	19
Educational	17	15	8	4
Governmental	15	10	8	2
Religious	9	6	4	3
Industrial	3	2	0	0

**FIGURE 9:**
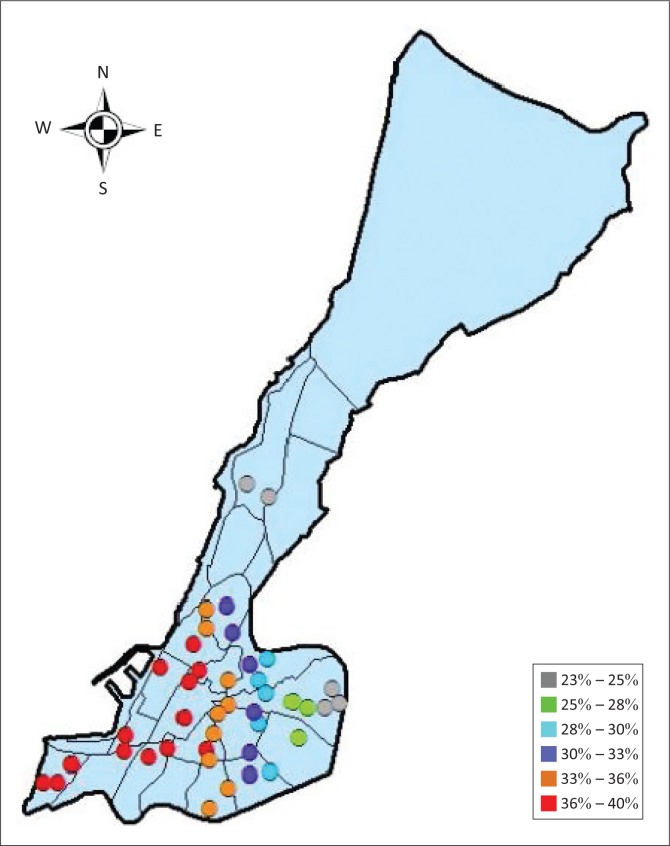
Moderately damaged schools.

In terms of educational buildings, schools would be the most affected by a medium earthquake scenario, with probably four schools completely damaged and eight schools extensively damaged; this can cause significant casualties if an earthquake occurs during the day. Among the essential facilities, emergency response will be affected since the city has an older hospital that was built before 1960. The results also show that this hospital would be extensively damaged if an earthquake of 5.5 Mw occurs. Figure 9 shows the distribution of moderately damaged schools.

#### Building damage by construction year

**FIGURE 10:**
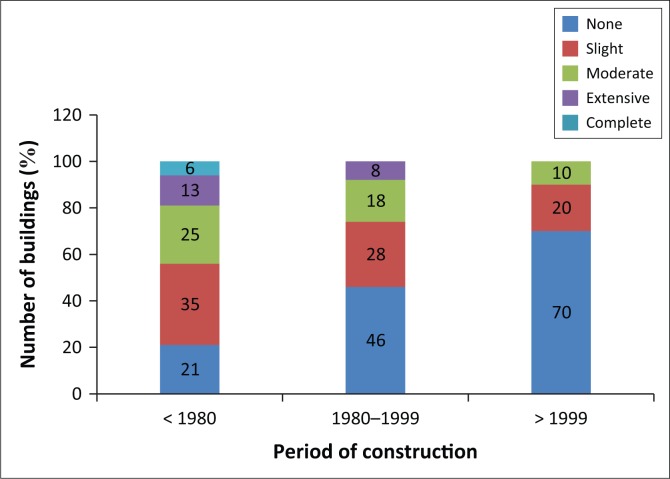
Building damage for different built years.

Knowing that Mostaganem city contains a significant number of buildings built before 1980 (the date of establishment of the first Algerian regulation), the majority of those structures were constructed without seismic regulations, which explains why 6% of buildings built before 1980 for both concrete frame and URM are estimated to collapse; 13% were extensively damaged and 25% were moderately damaged under 5.5 Mw earthquake scenarios. After the strong earthquake that took place in the city of ‘Chlef’ on 10 October 1980, with a magnitude of 7.2 Mw, the Algerian Seismic Regulation was revised, which led to the introduction of new methods for calculating seismic force in structures. In this regard, buildings constructed between 1980 and 1999 are more resistant, with only 8% of those structures extensively damaged and only 18% moderately damaged. The last range of buildings was built after 1999, after the latest revision of the Algerian Seismic Regulation following the strong earthquake that occurred on 21 May 2010 (6.8 Mw). Later, a new seismic zoning of the country was established, and stricter rules were adopted, which explains why no buildings built after 1999 were completely or extensively damaged – only 10% of these buildings were moderately damaged. Figure 10 summarises the percentage of building damage, categorised according to the three construction periods.

### Casualty loss

The HAZUS programme breaks down casualties into four injury severity levels, from those requiring basic medical aid to those involving severe injury which cause instantaneous death. In this study, the casualties during early morning hours, such as 02:00, is considered to be the worst–case scenario because it is assumed that all people are at home during these early hours. An analysis of the rate of casualty caused by URM buildings built before 1980 reveals that 10.5% of the residents of URM buildings are injured or dead. This result shows that URM is recognised as one of the most hazardous building types in terms of casualties in the study area. Compared with the URM, the concrete frame has lower casualty rates of 1.5% and 0.5% for those built before and after 1980, respectively.

## Conclusion

This work aims at evaluating the seismic vulnerability of the buildings in Mostaganem city, which has not been recognised as a high seismic zone in spite of having a large number of old buildings. For this reason, the city has not been subject to a seismic vulnerability assessment. Therefore, a lack of awareness and preparation for seismic hazards has created a scenario where even a medium earthquake of 5.5 Mw, with its epicentre inside the city, can lead to disastrous consequences. This was demonstrated in this study by establishing fragility curves for each building type, using non-linear APA and then inputting the parameters of those curves in the HAZUS software and modifying it to suit the typical conditions and parameters of the city of Mostaganem.

The results indicate that the number of poor-quality buildings expected to be totally destroyed could be more than 28, and the number of casualties could reach several hundreds. On the contrary, for high-quality structures there are no buildings that are expected to be completely destroyed.
